# Implementation of an electronic health record—based tool increases administration of venous thromboembolism chemoprophylaxis in trauma

**DOI:** 10.1016/j.surg.2025.109857

**Published:** 2025-11-17

**Authors:** Zongyang Mou, Parisa Oviedo, Louis Perkins, Todd W. Costantini, Jeanne G. Lee, Allison E. Berndtson, Laura N. Haines, Aaron Marshall, Jarrett E. Santorelli

**Affiliations:** a Department of Surgery, Division of Trauma, Surgical Critical Care, Burns and Acute Care Surgery, UC San Diego Health, San Diego, CA; b Department of Surgery, Division of Trauma, Division of Critical Care and Acute Care Surgery, University of Minnesota, Minneapolis, MN; c Department of Surgery, Division of Trauma and Acute Care Surgery, University of Colorado Hospital, Aurora, CO

## Abstract

**Introduction:**

Timely initiation of venous thromboembolism chemoprophylaxis remains a challenge in trauma and is recognized as a quality benchmark for the American College of Surgeons Trauma Quality Improvement Program. There are several workflow-related barriers that limit timely administration of venous thromboembolism chemoprophylaxis. Tools embedded within the electronic health record can aid protocol compliance as they are integrated in the care workflow. We hypothesized that implementing an electronic health record—based clinical decision support tool improves venous thromboembolism chemoprophylaxis administration in patients with trauma admission.

**Methods:**

We conducted a pre- and postintervention study in patients admitted to a level 1 trauma center with a hospital length of stay greater than 2 days from November 2018 to May 2021. An electronic health record tool updated in real time indicating venous thromboembolism chemoprophylaxis status was incorporated into the daily handoff list in February 2020. No other initiatives were implemented at this time. Outcomes were the change in percentage of patients receiving at least 1 venous thromboembolism chemoprophylaxis dose during hospitalization and time to first chemoprophylaxis dose.

**Results:**

There were 4,311 patients: 2,174 in the preintervention group and 2,137 in the postintervention group. The percentage of patients receiving venous thromboembolism chemoprophylaxis increased from 57.9% before intervention to 81.8% after intervention (*P* < .001). Concurrently, there was a decrease in average time to initiation of pharmacologic venous thromboembolism chemoprophylaxis from 2.2 days to 1.6 days (*P* < .001).

**Conclusion:**

We found that implementing an electronic health record—embedded tool increased the proportion of patients receiving venous thromboembolism chemoprophylaxis and decreased the time to first dose of venous thromboembolism chemoprophylaxis. This demonstrates the benefits of using electronic health record—based tools to support trauma protocol compliance.

## Introduction

Venous thromboembolism (VTE) is a significant cause of morbidity and mortality after trauma, affecting up to 63% of patients.^1–5^ Studies have demonstrated that early administration of chemoprophylaxis reduces VTE rates and associated mortality.^6,7^ Recent guidelines developed by the American Association for the Surgery of Trauma and the American College of Surgeons Committee on Trauma recommend prompt delivery of VTE chemoprophylaxis within the first 48 hours of hospitalization for most patients.^8^ Despite the well-known risks of VTE, appropriate adherence to chemoprophylaxis protocols for at-risk patients remains challenging.^8—10^ Trauma patients are at especially high risk of missed VTE doses given the need for frequent surgeries or procedures, which may require holding doses of chemoprophylaxis, elevated bleeding risk (both real and perceived), and higher medical complexity, particularly among those with polytraumatic injuries.^11—14^ Many trauma patients experience interruptions in dosing, which leads to a significantly higher risk of VTE.^15—17^

Recent efforts focused on leveraging electronic health record (EHR) systems have increased VTE chemoprophylaxis adherence.^18^ Haut et al^19^ developed one of the first trauma-specific computerized clinical decision support (CDS) systems within the EHR that used a checklist of risk factors to recommend appropriate prophylaxis, which significantly improved compliance. However, many tools and CDS such as these are developed for institution-specific EHRs and are limited in their ability to be widely disseminated and implemented.^20,21^ With the EHR marketplace becoming more sophisticated and concentrated to several major systems, leveraging prebuilt or native tools within the EHR to improve quality such as VTE chemoprophylaxis represents a valuable avenue for interventions.^22^ We thus hypothesized that implementing a prebuilt EHR-embedded VTE status tool that continuously tracks patient VTE chemoprophylaxis status would lead to higher rates of compliance with the VTE chemoprophylaxis protocol and would decrease the time to the first dose of chemoprophylaxis.

## Methods

### Cohort selection

We conducted a pre- and postintervention study at an urban level 1 trauma center from November 2018 to May 2021. The pre- and postintervention cohorts were defined as the 15-month period before and after implementation of our EHR tool on February 1, 2020. Patients were included in this study if they were an adult patient aged 18 years or older identified by the trauma registry with a hospital length of stay (LOS) greater than 48 hours. Our institution's default protocol for VTE chemoprophylaxis initiation is within 24 hours using weight-based low-molecular-weight heparin (enoxaparin) twice per day, unless there is a contraindication such as intracranial hemorrhage. At our institution, enoxaparin is the preferred agent instead of unfractionated heparin in trauma patients, and chemoprophylaxis is generally not held for operations or procedures except for neurological and spine operations.

There was no additional concurrent quality improvement project targeting VTE chemoprophylaxis adherence during the study period. This study was approved by the institutional review board of the University of California San Diego Human Research Protection Program (IRB #: 151611). A waiver of informed consent was also granted.

### EHR tool

Positive VTE chemoprophylaxis was defined as a prophylactic or therapeutic dose of heparin (subcutaneous or intravenous) or enoxaparin (subcutaneous). We used the prebuilt VTE chemoprophylaxis status tool developed by Epic Systems (Verona, WI) for their EHR as our intervention (Supplementary Figure). This tool detects whether VTE chemoprophylaxis has been ordered for a patient and whether the patient received a dose in the last 24 hours based on the medical administration record and displays this real-time information in the shared patient handoff, which is used by health care providers. This was not a pop-up alert, active alert, or best practice advisory trigger and instead was a passive tool that did not interrupt provider workflow and minimized alert fatigue. Given that this tool provided easy to understand binary graphical outputs, there was no additional staff training required before implementation. Trauma providers were informed when this tool was formally added to the patient list, which marked the beginning of the intervention study period.

### Variable and outcomes

The primary outcomes of this study were the percentage of patients who received at least 1 dose of VTE chemoprophylaxis during their hospitalization and the time (in days) to the first dose of VTE chemoprophylaxis from admission. Secondary outcomes were changes in VTE and pulmonary embolism rates. Covariates for our analysis were patient age, sex, body mass index (BMI), new injury severity scale (NISS), type of trauma (blunt versus penetrating), LOS, mortality, and presence of spleen, liver, spine, or head injury. We chose these specific organ injuries as they are the most common contraindications to VTE chemoprophylaxis initiation in the immediate postinjury phase. All data were obtained from the local trauma registry. Missing data were imputed using the multiple imputation method as previously described.^23^

### Statistical analysis

R Statistical Software (version 4.0.5; R Core Team, 2020; Vienna, Austria) was used for all statistical analysis. The χ^2^ test was used to compare differences for categorical variables such as event rates between the 2 cohorts. The 2-sample independent means *t* test was used for continuous variables. Multivariable logistic and linear regression models were used to evaluate our primary outcomes of interest. A *P* value of .05 was defined as the threshold of statistical significance.

Multivariable regression analysis was used to evaluate the impact of the EHR tool intervention while adjusting for relevant covariates. As detailed in Supplementary Table S1, a linear regression model was used to assess days from admission to first VTE chemoprophylaxis dose (Supplementary Table S1), whereas a logistic regression model (Supplementary Table S2) was used to assess receipt of any VTE chemoprophylaxis during hospital admission. Covariates included age, sex, BMI, Injury Severity Score, hospital LOS, and the presence of traumatic brain injury (TBI), spine injury, splenic trauma, or liver trauma.

## Results

In the 30-month study period, we had 7,099 patient admissions, of which 4,311 met inclusion criteria. There were 2,174 patients in the preintervention cohort and 2,137 patients in the postintervention cohort. Both groups had similar demographics, mortality, LOS, and injury severity and composition (Table). We found that after implementation of our EHR reporting tool, the percentage of patients receiving at least 1 dose of VTE chemoprophylaxis significantly increased by 23.9% (*P* < .001, Figure). Concurrently, the time to first dose of VTE chemoprophylaxis decreased from 2.2 days before intervention to 1.6 days after intervention (*P* < .001, Figure). Interrupted time series analysis showed at baseline; there was a small but significant increase in monthly VTE chemoprophylaxis administration (1.7% per month, *P* < .001). However, independent of this baseline increase, implementation of the intervention resulted in an additional increase of 11.1% in monthly VTE chemoprophylaxis administration (*P* < .001). As shown in the linear regression model (Supplementary Table S1), the EHR tool was associated with a significant reduction in time, 0.43 days, from admission to first chemoprophylaxis dose compared with the cohort without the tool (*P* < .001). Furthermore, the changes in VTE chemoprophylaxis adherence were immediate and sustained throughout the duration of the postintervention study period.

Of the patients receiving VTE chemoprophylaxis, 88% were given enoxaparin in both the pre- and postintervention cohorts. Our multivariable regression model showed that while having a TBI, liver, spine, or spleen injury on admission, all were associated with increased time to VTE chemoprophylaxis (*P* < .001 for all), only TBI was associated with also having a decreased likelihood of ever receiving VTE chemoprophylaxis (odds ratio = 0.24, *P* < .01). We did not observe a change in VTE rates (2.1% preintervention vs 2.7% postintervention, *P* = .10) or pulmonary embolism rates (0.2% vs 0.6%, *P* = .23) when adjusted for age, sex, intensive care unit LOS, NISS, BMI, and type and mechanism of injury (Supplementary Tables S1 and S2).

## Discussion

We found that implementing a prebuilt EHR-embedded tool that displays VTE chemoprophylaxis status in real time resulted in increased administration of VTE chemoprophylaxis as well as decreased time to first dose in trauma patients. Although there was already a steady increase in VTE chemoprophylaxis administration rate before the study, the implementation of our tool enhanced that rate by 10-fold. The changes from our intervention were sustained throughout the postintervention period of our study. Thus, our results suggest that introducing this simple and passively displayed EHR tool was effective in improving VTE chemoprophylaxis adherence in trauma patients. In addition, although we found that patients in the intervention cohort had slightly longer hospital LOS, LOS was not significantly different between cohorts when adjusted for in our multivariable models.

Our results are in line with previous studies that have demonstrated significant efficacy in using EHR-based tools to improve VTE chemoprophylaxis adherence among trauma and surgical patients.^18,19,24^ However, our study differs in that it uses a prebuilt tool that displays real-time VTE information passively within the EHR workflow. This is a departure from traditional CDS support systems that mandate users to input specific patient information to generate guidance on whether a patient requires VTE chemoprophylaxis. Although such active CDS systems are effective, they run the risk of being counterproductive by increasing alert fatigue among clinicians and prompting users to either ignore or attempt work arounds.^25,26^ Conversely, passive CDS alerts may be missed by providers, which, in other studies, has limited their effectiveness.^27^

Although prior studies have suggested that passive EHR alerts are often missed or ignored by providers, limiting their effectiveness in changing clinical behavior, our findings suggest otherwise.^25,26^ We demonstrated a significant improvement in VTE chemoprophylaxis adherence after the implementation of as passive tool at our institution. This contrast may be explained by how the tool was integrated into provider workflow. Unlike passive alerts placed in ancillary EHR tabs or dashboards, our tool was embedded directly within the daily patient list used by trauma providers during rounds and handoffs. This nonoptional, high-visibility placement likely contributed to its effectiveness. Our findings align with more recent evidence suggesting that, when properly implemented, passive tools can indeed influence provider behavior. Goren et al^28^ demonstrated that passive EHR prompts significantly increased lipid screening rates in pediatric patients. Our study similarly showed improved adherence to VTE chemoprophylaxis protocols after implementation of a passive EHR tool. By embedding the tool in a high-visibility, nonoptional area, rather than a peripheral tab or dashboard, we likely optimized provider engagement. These findings suggest that passive CDS tools, although often dismissed as ineffective, can be impactful when strategically placed within mandatory workflows.

An additional advantage of our intervention is that it is a prebuilt tool developed by the EHR vendor and thus can be rapidly deployed across any health system that uses the same EHR. This allows it to bypass a multitude of barriers to implementation compared with externally built CDS tools including compliance with local EHR security protocols, the need for an in-house expert with deep knowledge of the tool and health system, data compatibility, and local system upkeep and monitoring.^21^ Another strength of our tool is its content-agnostic design. Rather than encoding specific clinical recommendations that require frequent updates as guidelines evolve, the tool simply indicates whether it has been ordered. This approach avoids the need for constant revision in response to minor changes in best practices—such as adjustments to enoxaparin dosing based on emerging studies—and ensures sustained relevance over time. Furthermore, the financial cost of implementation is minimal compared with the need for external CDS tools. Given the ongoing variations in VTE chemoprophylaxis adherence rates among centers across the country, this tool's scalability and low cost are significant advantages over externally developed VTE chemoprophylaxis CDS systems.^8^

Our study has several limitations that should be considered when interpreting the results. First, this is a single institution study, and its benefit may not be as significant at other institutions depending on a multitude of factors including baseline compliance rate, institutional culture, and other quality improvement initiatives. Second, we do not have the previously described CDS systems at our institution to perform a comparison study and evaluate whether one tool is more effective than the other. Third, we did not observe a difference in VTE and PE rates, although these were low at baseline and our study did not have sufficient power to detect a difference. In addition, VTE chemoprophylaxis reduces but does not eliminate the risk of these events.^29^ Furthermore, although this tool is readily available in the Epic EHR, some finetuning would be required at each local institution to ensure accuracy. Lastly, although the EHR tool was associated with a significant increase in VTE chemoprophylaxis rate, future studies should also collect data on dose-by-dose adherence or incidence of missed doses during the hospital stay.

In conclusion, implementing a prebuilt EHR tool that passively displays VTE chemoprophylaxis status in real time improves protocol adherence and decreases time to first dose. Trauma centers with the appropriate EHR and access to this tool should consider its implementation to assist with improving and maintaining VTE chemoprophylaxis adherence.

## Supplementary Material

MMC1

figs1

Supplementary material associated with this article can be found, in the online version, at [https://doi.org/10.1016/j.surg.2025.109857].

## Figures and Tables

**Figure.**
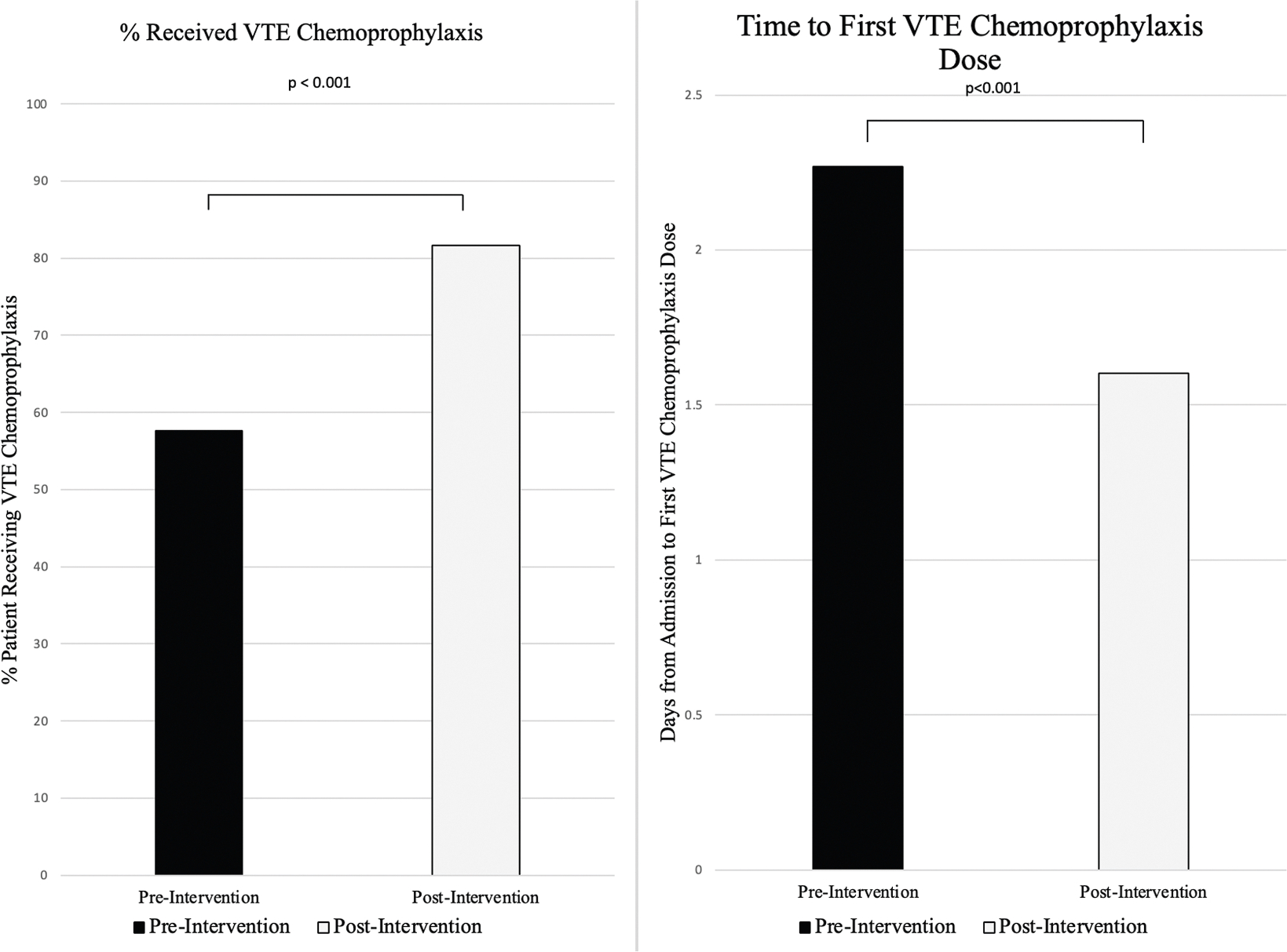
(A) Percentage of patients in the cohort who received at least 1 dose of VTE chemoprophylaxis. The preintervention cohort had 57.9% patients, and the postintervention cohort had 81.8% (*P* < .001). (B) Time from admission to the first dose of VTE chemoprophylaxis in days. The preintervention cohort had a time of 2.2 days, and the postintervention cohort had 1.6 days (*P* < .001). *VTE*, venous thromboembolism.

**Table:** Characteristics of the pre- and postintervention cohorts

Characteristic	Preintervention (*N* = 2,174)	Postintervention (*N* = 2,137)	*P* value

Age (yr)	53.9	52.4	.03
Sex (male) (%)	68.2	69.4	.40
Mortality (%)	2.6	2.6	.99
Median LOS (d)	4.0	4.0	.12
Median ICU LOS (d)	2.4	3.1	<.01
Mean NISS	14.7	15.7	.01
Mean BMI	29.2	27.4	<.01
Blunt trauma (%)	7.6	9.2	.07
Head trauma (%)	25.5	24.3	.38
Liver trauma (%)	2.4	2.6	.70
Spine trauma (%)	20.8	22.7	.14
Spleen trauma (%)	2.2	2.2	.98

*BMI*, body mass index; *ICU*, intensive care unit; *LOS*, length of stay; *NISS*, new injury severity scale.
