# Supplementary material for: Implementation of an electronic health record—based tool increases administration of venous thromboembolism chemoprophylaxis in trauma
Source: Surgery. Author manuscript; Available in PMC 2026 Aug 4. (PMC13435931; doi:10.1016/j.surg.2025.109857)
Supplement: MMC1 [file NIHMS2186259-supplement-MMC1.docx]

# Supplementary Table S1. Multivariable Linear Regression Model Adjusting for Time to First Dose of Chemoprophylaxis in Days

| Variable | Coefficient | Std. Error | t-value | 95% CI Lower | 95% CI Upper | P-value |
| --- | --- | --- | --- | --- | --- | --- |
| (Intercept) | 0.538 | 0.132 | 4.073 | 0.279 | 0.796 | 0.0 |
| Length of Stay | 0.001 | 0.001 | 1.17 | -0.001 | 0.003 | 0.242 |
| ICU Length of Stay | 0.003 | 0.003 | 1.189 | -0.002 | 0.008 | 0.235 |
| Intervention | -0.727 | 0.151 | -4.818 | -1.023 | -0.431 | 0.0 |
| Injury Type | -0.074 | 0.052 | -1.427 | -0.175 | 0.027 | 0.154 |
| Diagnosis of Traumatic Brain Injury | 0.658 | 0.042 | 15.807 | 0.577 | 0.74 | 0.0 |
| Diagnosis of AIS Spine Injury | 0.151 | 0.036 | 4.258 | 0.082 | 0.221 | 0.0 |
| Diagnosis of Splenic Trauma | 0.37 | 0.095 | 3.898 | 0.184 | 0.556 | 0.0 |
| Diagnosis of Liver Trauma | 0.251 | 0.089 | 2.832 | 0.077 | 0.425 | 0.005 |
| BMI | 0.003 | 0.001 | 2.208 | 0.0 | 0.006 | 0.027 |
| Injury Severity Score (ISS) | 0.008 | 0.002 | 4.951 | 0.005 | 0.011 | 0.0 |

# Supplementary Table S2. Multivariable Logistic Regression Model, in Received Chemoprophylaxis

| Variable | Coefficient | Std. Error | z-value | 95% CI Lower | 95% CI Upper | P-value |
| --- | --- | --- | --- | --- | --- | --- |
| (Intercept) | -2.356 | 0.393 | -5.995 | -3.127 | -1.586 | 0.0 |
| Length of Stay | 0.311 | 0.027 | 11.492 | 0.258 | 0.364 | 0.0 |
| ICU Length of Stay | -0.054 | 0.038 | -1.441 | -0.128 | 0.02 | 0.15 |
| Intervention | 2.686 | 0.497 | 5.402 | 1.711 | 3.661 | 0.0 |
| Injury Type | -0.042 | 0.194 | -0.215 | -0.423 | 0.339 | 0.83 |
| Diagnosis of Traumatic Brain Injury | -1.454 | 0.131 | -11.096 | -1.711 | -1.197 | 0.0 |
| Diagnosis of AIS Spine Injury | 0.085 | 0.14 | 0.604 | -0.19 | 0.36 | 0.546 |
| Diagnosis of Splenic Trauma | -0.081 | 0.454 | -0.177 | -0.969 | 0.808 | 0.859 |
| Diagnosis of Liver Trauma | -0.02 | 0.427 | -0.046 | -0.857 | 0.817 | 0.963 |
| BMI | 0.003 | 0.004 | 0.654 | -0.006 | 0.012 | 0.513 |
| Injury Severity Score (ISS) | 0.018 | 0.006 | 3.014 | 0.006 | 0.031 | 0.003 |

*Where AIS = abbreviated injury score , BMI = body mass index, NISS = new injury severity score
