# Supplementary figures and images for: Implementation of an electronic health record—based tool increases administration of venous thromboembolism chemoprophylaxis in trauma
Source: Surgery. Author manuscript; Available in PMC 2026 Aug 4. (PMC13435931; doi:10.1016/j.surg.2025.109857)

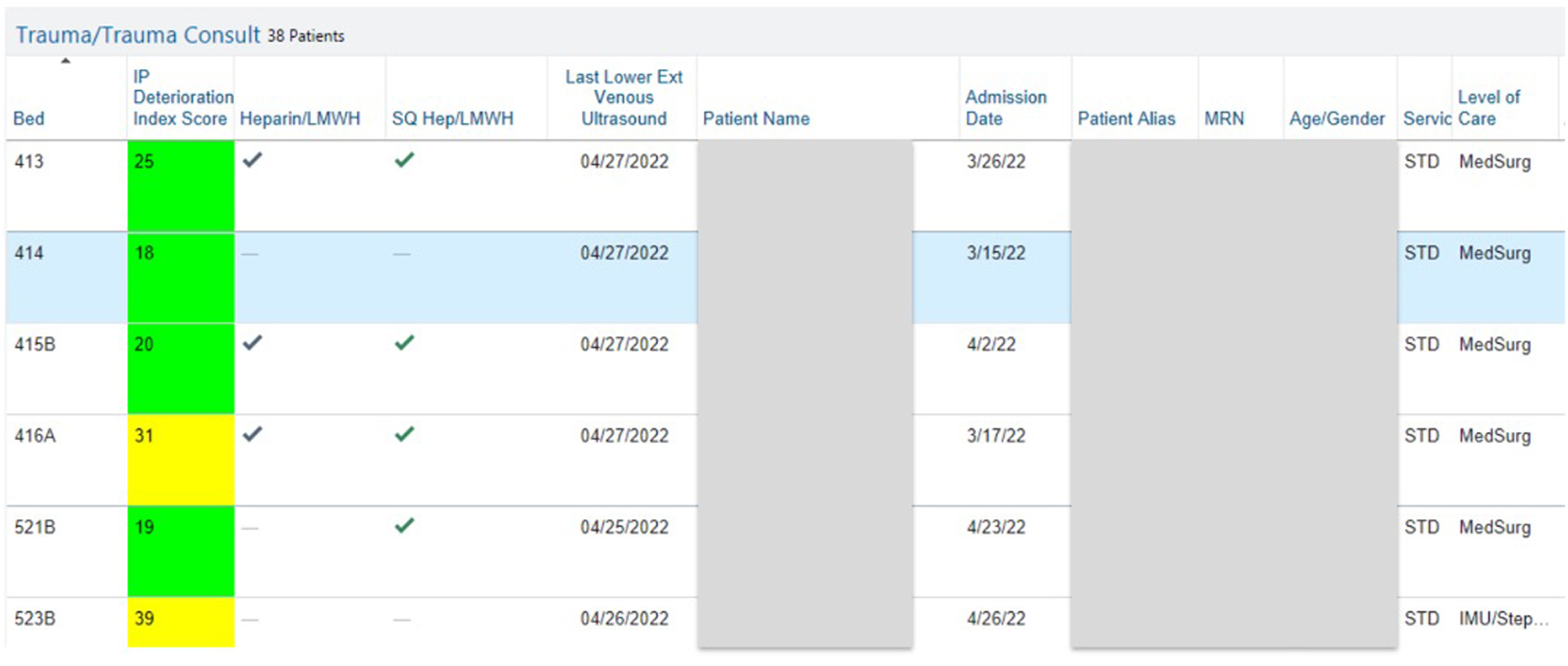

Supplement: figs1 [file NIHMS2186259-supplement-figs1.jpg]
